# Exception Sets of Intrinsic and Piecewise Lipschitz Functions

**DOI:** 10.1007/s12220-021-00860-5

**Published:** 2022-02-01

**Authors:** Gunther Leobacher, Alexander Steinicke

**Affiliations:** 1 Institute of Mathematics and Scientific Computing, University of Graz, Heinrichstraße 36, 8010 Graz, Austria; 2 Institute of Mathematics, University of Rostock, Ulmenstraße 69, Haus 3, 18051 Rostock, Germany; 3 Institute of Applied Mathematics, Montanuniversitaet Leoben, Peter-Tunner-Straße 25/I, 8700 Leoben, Austria

**Keywords:** Intrinsic metric, Permeable sets, Piecewise Lipschitz continuity, 54C05, 54E40

## Abstract

We consider a class of functions defined on metric spaces which generalizes the concept of piecewise Lipschitz continuous functions on an interval or on polyhedral structures. The study of such functions requires the investigation of their exception sets where the Lipschitz property fails. The newly introduced notion of permeability describes sets which are natural exceptions for Lipschitz continuity in a well-defined sense. One of the main results states that continuous functions which are intrinsically Lipschitz continuous outside a permeable set are Lipschitz continuous on the whole domain with respect to the intrinsic metric. We provide examples of permeable sets in ${{\mathbb {R}}}^d$, which include Lipschitz submanifolds.

## Introduction

Exception sets for the regularity of a function are encountered when considering functions $f:I\rightarrow {{\mathbb {R}}}$ defined on an interval *I* that have a certain property (e.g. continuity, differentiability) when restricted to a subset $E\subseteq I$ but not on the whole of *I*. The complement $\Theta =I\setminus E$ is then an *exception set* for the function’s property. A particularly common example here is the case of a finite exception set $\Theta$ for a function defined on a real interval. Such a finite set partitions the interval *I* into several parts or *pieces*, hence leading to the notions of *piecewise* continuous, differentiable, etc. functions. The property which will be of our interest is the one of Lipschitz continuity and our work is motivated by the desire to generalize the notion of ‘piecewise Lipschitz continuity’ to the multidimensional case. Consider the following definition:

### Definition 1

A function $f:I\rightarrow {{\mathbb {R}}}$ is *piecewise Lipschitz continuous* if there exist finitely many points $x_1,\ldots ,x_n\in I$ with $x_0:=\inf I<x_1<\ldots <x_{n+1}:=\sup I$, such that $f|_{(x_{j-1},x_j)}$ is Lipschitz continuous for every $j=1,\dots ,n+1$.

With this definition, the following result is easily proven (see, for example, Lemma 2.4 in [18]):

### Lemma 2

Let $I\subseteq {{\mathbb {R}}}$ be an interval and let $f:I\rightarrow {{\mathbb {R}}}$ be continuous and piecewise Lipschitz continuous. Then *f* is Lipschitz continuous.

Definition 1 is a reasonable implementation of the concept, although we do not claim that it is universally accepted across the mathematical community. Generalizing this (or a similar) definition to the multidimensional case is far from being unambiguous as many multidimensional concepts coincide in dimension 1: Intervals are precisely the convex subsets of ${{\mathbb {R}}}$, but also precisely the star-shaped, connected, path-connected, arc-connected sets and the polytopes. Multidimensional generalizations of the exception set $\{x_1,\ldots ,x_n\}$ in Definition 1 are affine hyperplanes, (finite unions of) submanifolds, finite sets, among others. Classical generalizations of Definition 1 to higher dimensions require the desired property on elements of a polytopal, polyhedral or simplicial subdivision of the domain. Variants of this procedure are well known for defining the class of *piecewise linear* (pl) or *piecewise differentiable* (pdiff) functions, see e.g., [26, 1.4, Ch.1], [28, Section 2.2] or [32, Section 3.9]. However, these classes comprise of continuous functions. A definition of piecewise linear using a subdivison by hyperplanes not implying continuity can be found in the introduction of [8]. A simple extension of the notion ‘piecewise Lipschitz continuous’, loosely following the ideas in the references above, could be given by the subsequent definition:

### Definition 3

Let $M\subseteq {{\mathbb {R}}}^d$. A function $f:M\rightarrow {{\mathbb {R}}}$ is *piecewise Lipschitz continuous* on *M* if there exist finitely many open polyhedra^1^$P_1,\ldots ,P_m$ with $P_j\cap P_k=\emptyset$ for $j\ne k$ and $M\subseteq \bigcup _{j=1}^n {\overline{P}}_j$, such that $f|_{P_j}$ is Lipschitz continuous for every $j=1,\dots ,n$.

The definition in [22] (implicit in Assumption 2.2) is similar to Definition 3, but replaces ‘open polyhedra’ by ‘open sets’. In [4], general sets for the $P_i$ are allowed, and the resulting difficulties are overcome by considering a ‘dispersed’ family of functions which means that not too many of them have their Lipschitz-exceptions around the same spot. We will take a path different from Definition 3 by concentrating on a notion which, instead of focussing on the *pieces* on which the Lipschitz property holds, we emphasize the *exception set* where the Lipschitz property fails. Related approaches can be found in [19], where the authors consider functions which are Lipschitz on each of two parts of a domain which is split by a $C^{1,1}$ manifold. A more general exception set occurs in [13, Section 14.2], where a piecewise Lipschitz continuous function is one which is is defined on a union of domains with Lipschitz boundaries, which is Lipschitz continuous on these subdomains. An even more complex exception set is allowed in [20], where a function is ‘piecewise $C^2$’, if the $C^2$ property fails on a closed set of Lebesgue-measure 0. An interesting theorem outside the ${{\mathbb {R}}}^d$-setting can be found in [7], where it is shown that a specific notion of piecewise Lipschitz continuity follows from a local Lipschitz condition for semi-algebraic functions ${{\mathbb {Q}}}_p^d\rightarrow {{\mathbb {Q}}}_p$, where ${{\mathbb {Q}}}_p$ are the *p*-adic numbers.

A guiding principle for what we attempt here will be that a suitable generalization of Lemma 2 should hold. We generalize and extend the approach of [18], who call a function $f:{{\mathbb {R}}}^d\rightarrow {{\mathbb {R}}}^m$ *piecewise Lipschitz* with exception set $\Theta \subseteq {{\mathbb {R}}}^d$, if $f|_{{{\mathbb {R}}}^d\setminus \Theta }$ is Lipschitz with respect to the intrinsic metric (see Definition 6) on ${{\mathbb {R}}}^d\setminus \Theta$, and where $\Theta$ is a hypersurface, that is, a $(d-1)$-dimensional submanifold of ${{\mathbb {R}}}^d$. They prove a multidimensional version of Lemma 2 under an additional condition on $\Theta$, namely, the condition that we will call *finitely permeable* (see Definition 10). The task to determine suitable exception sets for Lipschitz functions with respect to the intrinsic metric should not be mixed with the—of course related—problem of finding sets *R* such that functions defined on the complement $R^c$ and belonging to a certain regularity class there may be extended to the whole space. Such *removable* sets *R* have been investigated in complex analysis and geometric function theory for a long time, see e.g., [15, 36], and, in connection with Lipschitz continuity, [9]. In the early account [1], removable sets are called *function theoretic null-sets* and are characterized by an extremal distance condition.

The generalization of piecewise Lipschitz continuous functions to Lipschitz continuous functions with respect to the intrinsic metric up to an exception set includes far more functions than when the induced metric on the complement of the exception is used, see Example 8 in Sect. 2 for instance. Also, Lemma 2 does not simply follow from well-known extension theorems, such as the classical ones of Kirszbraun (see [17, 29, p.21]), McShane-Whitney (see [21, 34]) or more recent ones such as the one in [23].

We expand the generalization from [18] in many directions. In Sect. 2 we first recall the notion of the intrinsic metric and introduce the concept of permeable and finitely permeable sets. This is done in the general framework of metric spaces. We define intrinsically Lipschitz continuous functions as functions which are Lipschitz continuous with respect to the intrinsic metric on their domain. Our first main result is then Theorem 15, which is a multidimensional version of Lemma 2, where the additional assumption is that the exception set is permeable. No further assumptions are required, in particular, the exception set need not be a manifold. The proof uses transfinite induction and the Cantor-Bendixson theorem.

The notion of permeability is weaker than that of finite permeability, and much weaker than finiteness, and we show that in the 1-dimensional real case it not only is a sufficient condition on a set $\Theta$ so that every continuous function which is Lipschitz continuous with exception set $\Theta$ is Lipschitz—it is also necessary.

Section 3 is then dedicated to finding large and practically relevant classes of subsets of ${{\mathbb {R}}}^d$ which are permeable. We show in Theorem 31 that every Lipschitz submanifold which is a closed subset of ${{\mathbb {R}}}^d$ is finitely permeable and thus permeable. We discuss further generalizations, instructive examples and counterexamples.

Our research presents a new concept in analysis with already a number of non-trivial results and generalizations. Moreover, it opens pathways to generalizing results in many applied fields, where concepts of piecewise Lipschitz continuous functions have already been used, such as image processing [5], uncertain input data problems [13], optimal control [14], stochastic differential equations [18], information processing [6, 25], machine learning [4, 30], dynamical systems [31], shape-from-shading problems [33].

## Intrinsic Lipschitz Functions and Permeable Subsets of Metric Spaces

Throughout this section, let (*M*, *d*) be a metric space. To begin, we recall some definitions for metric spaces.

### Definition 4

(*Path, arc, length*)

1. A *path* in *M* is a continuous mapping $\gamma :[a,b]\rightarrow M$. We also say that $\gamma$ is a path in *M* from $\gamma (a)$ to $\gamma (b)$.
2. An injective path is called an *arc*.
3. If $\gamma :[a,b]\rightarrow M$ is a path in *M*, then its *length* $\ell (\gamma )$ is defined as
  $$\begin{aligned} \ell (\gamma ) :=\sup \Big \{\sum _{k=1}^n d\big (\gamma (t_k),\gamma (t_{k-1})\big ):n\in {{\mathbb {N}}},\,a=t_0<\ldots <t_n=b\Big \}\,. \end{aligned}$$

The following important lemma is an immediate consequence of Propositions 3.4 and 3.5 in [2]. It states that one can always replace a path in *M* by an injective one with length at most that of the original path and its image contained in that of the original path.

### Lemma 5

Let $x,y\in M$, $x\ne y$ and $\gamma :[0,1]\rightarrow M$ be a path from *x* to *y*. Then there exists an arc $\eta :[0,1]\rightarrow M$ from *x* to *y* with $\eta ([0,1])\subseteq \gamma ([0,1])$ and $\ell (\eta )\le \ell (\gamma )$.

### Proof

Consider first the case where $\gamma$ has finite length. Then $\gamma ([0,1])$ is a continuum and by [2, Proposition 3.5]

$$\begin{aligned} \ell (\gamma )=\int _{\gamma ([0,1])}m(\gamma ,x)d{\mathscr {H}}^1(x)\ge \int _{\gamma ([0,1])}d{\mathscr {H}}^1(x) = {\mathscr {H}}^1\big (\gamma ([0,1])\big )\,, \end{aligned}$$

where $m(\gamma ,.)$ is the multiplicity of $\gamma$, $m(\gamma ,x):=\#\big (\gamma ^{-1}(\{x\})\big )$, and ${\mathscr {H}}^1$ is the 1-Hausdorff-measure. Now by [2, Proposition 3.4], $\gamma (0)$ and $\gamma (1)$ are connected by an arc $\eta$ in $\gamma ([0,1])$ with $\ell (\eta )\le {\mathscr {H}}^1\big (\gamma ([0,1])\big )\le \ell (\gamma )$.

If $\ell (\gamma )=\infty$, the assertion follows immediately from the Hahn-Mazurkiewicz theorem, see [35, Section 31]. $\square$

### Definition 6

(*Intrinsic metric, length space, quasi-convexity*) Let $E\subseteq M$ and $\Gamma (x,y)$ be the set of all paths of finite length in *E* from *x* to *y*. The *intrinsic metric* $\rho _E$ on *E* is defined by

$$\begin{aligned} \rho _E(x,y):=\inf \big \{\ell (\gamma ):\gamma \in \Gamma (x,y)\big \}\,, \qquad (x,y\in E)\,, \end{aligned}$$

with the convention $\inf \emptyset =\infty$. The metric space (*M*, *d*) is a *length space* iff $\rho _M=d$. We call (*M*, *d*) *C*-*quasi-convex* iff there exists $C>0$ s.t. $\rho _M(x,y)\le C d(x,y)$ for all $x,y\in M$.

Note that $\rho _E$ is not a proper metric in that it may assume the value infinity. Of course, one could relate $\rho _E$ to a proper metric

$$\begin{aligned} {\tilde{\rho }}_E (x,y) :={\left\{ \begin{array}{ll} \frac{\rho _E(x,y)}{1+\rho _E(x,y)}\,, &{}\text {if } \rho _E(x,y)<\infty \,,\\ 1\,, &{}\text {if } \rho _E(x,y)=\infty \,. \end{array}\right. } \end{aligned}$$

However, we stick to $\rho _E$, as it is the more natural choice and the extended co-domain does not lead to any difficulties.

It is readily checked that, if we allow $\infty$ as the value of a metric, then $(E,\rho _E)$ is a length space.

See [12, Section 7] and [11] for interesting consequences of quasi-convexity in the context of Lipschitz analysis.

### Definition 7

(*Intrinsically Lipschitz continuous function*) Let $E\subseteq M$, let $(Y,d_Y)$ be a metric space and $f:M\rightarrow Y$ a function.

1. We call *f* *intrinsically* *L*-*Lipschitz continuous* on *E* iff $f|_E:E\rightarrow Y$ is Lipschitz continuous with respect to the intrinsic metric $\rho _E$ on *E* and $d_Y$ on $Y$ and Lipschitz constant *L*.
2. We call *f* *intrinsically Lipschitz continuous* on *E* iff *f* is intrinsically *L*-Lipschitz continuous for some *L*.
3. In the above cases we call $M\setminus E$ an *exception set (for intrinsic Lipschitz continuity)* of *f*.

### Example 8

Consider the function $f:{{\mathbb {R}}}^2\longrightarrow {{\mathbb {R}}}$, $f(x)=\Vert x\Vert \arg (x)$. Then *f* is not Lipschitz continuous with respect to the induced metric, since $\lim _{h\rightarrow 0+}f(\cos (\pi -h),\sin (\pi -h))=-\pi$ and $\lim _{h\rightarrow 0+}f(\cos (\pi +h),\sin (\pi +h))=\pi$.

It is readily checked, however, that *f* is Lipschitz continuous on $E={{\mathbb {R}}}^2\backslash \{x\in {{\mathbb {R}}}^2: x_1<0,x_2=0\}$ w.r.t. the intrinsic metric $\rho _E$ (note that *E* is not quasi-convex).

Thus *f* is intrinsically Lipschitz continuous with exception set $\Theta :=\{x\in {{\mathbb {R}}}^2: x_1<0,x_2=0\}$ in the sense of Definition 7.

The function $g:{{\mathbb {R}}}^2\longrightarrow {{\mathbb {R}}}$, $f(x)=\Vert x\Vert ^2\arg (x)$ is only locally intrinsically Lipschitz continuous on *E*.

A classical method for proving Lipschitz continuity of a differentiable function also works for intrinsic Lipschitz continuity:

### Example 9

Let $A\subseteq {{\mathbb {R}}}^d$ open and let $f:A\rightarrow {{\mathbb {R}}}$ be differentiable with $\sup _{x\in A}\Vert \nabla f(x) \Vert <\infty$. Then *f* is intrinsically Lipschitz continuous on *A* with Lipschitz constant $\sup _{x\in A}\Vert \nabla f(x) \Vert$.

A proof can be found in [18, Lemma 3.6].

It is almost obvious, that a function $f:{{\mathbb {R}}}^2\rightarrow {{\mathbb {R}}}$, which is continuous and intrinsically Lipschitz continuous on ${{\mathbb {R}}}^2\setminus \{(x_1,x_2):x_1<0, x_2=0\}$, is Lipschitz continuous in ${{\mathbb {R}}}^2$. One can use that $\Theta :=\{(x_1,x_2):x_1<0, x_2=0\}$ does not pose a ‘hard’ barrier, since every straight line connecting two points in ${{\mathbb {R}}}^2\setminus \Theta$ has at most one intersection point with $\Theta$ and so one can conclude the Lipschitz continuity by approaching $\Theta$ from either side (we invite the reader to make this argument rigorous – such a kind of argument will be used also in the proof of Theorem 15).

To make the elementary property of ‘not being a hard barrier’ precise, we define at this point the notion of permeability.

### Definition 10

Let $$E,\Theta \subseteq M$$.

1. The $\Theta$-*intrinsic metric* $\rho ^\Theta _E$ on *E* is defined by
  $$\begin{aligned} \rho ^\Theta _E(x,y):=\inf \big \{\ell (\gamma ):\gamma \in \Gamma ^\Theta (x,y)\big \} \, \end{aligned}$$
  where $\Gamma ^\Theta (x,y)$ is the set of all paths $\gamma :[a,b]\rightarrow M$ of finite length in *E* from *x* to *y*, such that $\overline{\{\gamma (t):t\in {[a,b]}\}\cap \Theta }$ is at most countable. (Again, we use the convention that $\inf \emptyset =\infty$.)
2. The $\Theta$-*finite intrinsic metric* $\rho ^{\Theta ,\textsc {fin}}_E$ on *E* is defined by
  $$\begin{aligned} \rho ^{\Theta ,\textsc {fin}}_E(x,y):=\inf \big \{\ell (\gamma ):\gamma \in \Gamma ^{\Theta ,\textsc {fin}}(x,y)\big \} \, \end{aligned}$$
  where $\Gamma ^{\Theta ,\textsc {fin}}(x,y)$ is the set of all paths $\gamma :[a,b]\rightarrow M$ of finite length in *E* from *x* to *y*, such that $\{\gamma (t):t\in {[a,b]}\}\cap \Theta$ is finite.
3. We call $\Theta$ *permeable relative to* *M* iff $\rho _{M}=\rho _M^\Theta$.
4. We call $\Theta$ *finitely permeable relative to* *M* iff $\rho _{M}=\rho _M^{\Theta ,\textsc {fin}}$.

When the ambient space (*M*, *d*) is understood and there is no danger of confusion, we simply say $\Theta$ is (finitely) permeable.

### Remark 11

A set $\Theta \subseteq M$ is (finitely) permeable iff for any $x,y\in M$ and every $\varepsilon >0$ there exists a path $\gamma$ from *x* to *y* in *M* with $\ell (\gamma )<\rho _M(x,y)+\varepsilon$ and such that $\overline{\{\gamma (t):t\in {[a,b]}\}\cap \Theta }$ is at most countable (finite). Clearly, every finitely permeable set is permeable.

The notion of permeability is related to that of metrical removability [15, Definition 1.1]: A set $\Theta \in M$ is *metrically removable* if for all $x,y\in M$ and all $\varepsilon >0$ there exists a path $\gamma$ in $(M\setminus \Theta )\cup \{x,y\}$ from *x* to *y* with $\ell (\gamma )<d(x,y)+\varepsilon$. Since $d(x,y)\le \rho (x,y)$ it follows that every metrically removable set is finitely permeable.

Lemma 3.7 in [15] states that if $M\subseteq {{\mathbb {R}}}^n$, then a subset $\Theta \subseteq M$ is metrically removable if and only if $\rho _M=\rho _{M\setminus \Theta }$. Therefore, for subsets *M* of the ${{\mathbb {R}}}^n$ with $\rho _M=d$ (i.e., *M* is a length space), metrical removability corresponds to Definition 10, where ‘countable’ or ‘finite’ is replaced by ‘empty’.

### Proposition 12

Let $\#M\ge 2$ and $\rho _M(x,y)<\infty$ for all $x,y\in M$. If $\Theta \subseteq M$ is permeable, then it has no interior point with respect to the original metric *d*.

### Proof

Let *x* be an interior point of $\Theta$ and $y\in M\setminus \{x\}$. By our assumption there exists a path $\gamma :[0,1]\rightarrow M$ from *x* to *y* with finite length. By Lemma 5 there exists an arc $\eta :[0,1]\rightarrow \gamma ([0,1])$ from *x* to *y* with $\ell (\eta )\le \ell (\gamma )$. Since *x* is an interior point of $\Theta$, there exists $r>0$ such that the ball $B_r(x):=\{y\in M: d(x,y)<r\}\subseteq \Theta$. By the continuity of $\eta$ there exists $\delta >0$ such that $\eta ([0,\delta ])\subseteq B_r(x)\subseteq \Theta$. Thus $\eta ([0,\delta ])\subseteq \{\gamma (t):t\in {[0,1]}\}\cap \Theta$, such that the latter set is uncountable. Since $\gamma$ was an arbitrary path in *M* from *x* to *y*, it follows that $\Gamma ^\Theta (x,y)=\emptyset$, and therefore $\rho _M^\Theta (x,y)=\infty >\rho _M(x,y)$. $\square$

We will show in Sect. 3 that all sufficiently regular sub-manifolds of the ${{\mathbb {R}}}^d$ of dimension smaller than *d* are finitely permeable. Next we show that (finite) permeability transfers to subsets.

### Proposition 13

Let $\Theta _0\subseteq \Theta \subseteq M$. If $\Theta$ is (finitely) permeable, then $\Theta _0$ is (finitely) permeable.

### Proof

Let $x,y\in M$ and $\varepsilon >0$. There exists $\gamma :[a,b]\rightarrow M$ such that $\ell (\gamma )<\rho _M(x,y)+\varepsilon$ and $\gamma ([a,b])\cap \Theta$ has countable closure. Since $\Theta _0\subseteq \Theta$, $\overline{\gamma ([a,b])\cap \Theta _0}\subseteq \overline{\gamma ([a,b])\cap \Theta }$. Therefore $\overline{\gamma ([a,b])\cap \Theta _0}$ is countable, hence $\rho ^{\Theta _0}_M(x,y)\le \rho _M(x,y)+\varepsilon$ from which the claim follows.

The ‘finitely permeable’ case follows from similar considerations. $\square$

### Proposition 14

Let $\Theta _0\subseteq \Theta \subseteq M$. If $\Theta$ is (finitely) permeable relative to (*M*, *d*) and $\Theta _0$ is closed in *M*, then $\Theta \setminus \Theta _0$ is (finitely) permeable relative to $(M\setminus \Theta _0,d)$.

### Proof

We only treat the permeable case, the finitely permeable one being almost identical.

Let $x,y\in M\setminus \Theta _0$ and let $\varepsilon >0$. If $\rho _{M\setminus \Theta _0}(x,y)=\infty$ there is nothing to show. Otherwise, there exists a path $\gamma :[0,1]\rightarrow M\setminus \Theta _0$ with $\gamma (0)=x$, $\gamma (1)=y$ and $\ell (\gamma )<\rho _{M\setminus \Theta _0}(x,y)+\frac{\varepsilon }{2}$. Since $\gamma ([0,1])$ is compact and $\Theta _0$ is closed in *M* there exists $\delta >0$ such that

$$\begin{aligned} \left\{ z\in M:\inf _{t\in [0,1]}d\big (\gamma (t),z\big )<\delta \right\} \cap \Theta _0=\emptyset \,. \end{aligned}$$

Next we can find $0=t_0<t_1<\dots <t_n=1$ such that $\rho _{M\setminus \Theta _0}(\gamma (t_{k-1}),\gamma (t_k))<\frac{\delta }{2}$ for all $k=1,\ldots ,n$. Since $\Theta$ is permeable relative to *M*, there exist $\eta _1,\ldots ,\eta _n:[0,1]\rightarrow M$ with $\eta _k(0)=\gamma (t_{k-1})$, $\eta _k(1)=\gamma (t_k)$,

$$\begin{aligned} \ell (\eta _k)&< \rho _M(\eta _k(0),\eta _k(1))+\min \left( \frac{\varepsilon }{2n},\frac{\delta }{2}\right) \\ \end{aligned}$$

and $\eta _k([0,1])\cap \Theta$ has countable closure for every $k\in \{1,\dots ,n\}$. Now

$$\begin{aligned} \ell (\eta _k)&< \rho _{M\setminus \Theta _0}(\gamma (t_{k-1}),\gamma (t_k))+\min \left( \frac{\varepsilon }{3n},\frac{\delta }{2}\right) <\delta \,, \end{aligned}$$

so that if $t\in [0,1]$, $d(\eta _k(t),\eta _k(0))\le \ell (\eta _k)< \delta$, and therefore $\eta _k(t)\notin \Theta _0$. Therefore $\eta :[0,1]\rightarrow M$, the concatenation of the paths $\eta _1,\ldots ,\eta _n$, is a path in $M\setminus \Theta _0$ with $\ell (\eta )<\ell (\gamma )+\frac{\varepsilon }{2}<\rho _{M\setminus \Theta _0}(x,y)+\varepsilon$ such that the closure of $\eta ([0,1])\cap (\Theta \setminus \Theta _0)$ is countable. $\square$

We now state our first main result.

### Theorem 15

Let $\Theta \subseteq M$ be permeable, $(Y,d_Y)$ a metric space. Then every continuous function $f:M\rightarrow Y$, which is intrinsically *L*-Lipschitz continuous on $E=M\setminus \Theta$, is intrinsically *L*-Lipschitz continuous on the whole of *M*.

For the proof of this result we use the following classical theorem, see for example [16, Theorem 6.11]:

### Theorem 16

(Cantor-Bendixson) Let *M* be polish. For every closed $A\subseteq M$ denote by *I*(*A*) are the isolated points in *A* and $H(A):=A\setminus I(A)$. For every ordinal $\alpha$ we set

$$\begin{aligned} H^{\alpha }(A):= {\left\{ \begin{array}{ll} A&{}\text {if } \alpha =0\,,\\ H\big (H^{\beta }(A)\big )\,, &{} \text {if } \alpha \text { is the successor of } \beta \,,\\ \bigcap _{\beta <\alpha } H^\beta (A)\,,&{} \text {if } \alpha \text { is a limit ordinal}\,. \end{array}\right. } \end{aligned}$$

Then for such a closed $A\subseteq M$ there exists a countable ordinal $\alpha _0$ such that for all $\alpha \ge \alpha _0:H^{\alpha }(A)=H^{\alpha _0}(A)$, i.e., $H^{\alpha _0}(A)$ is a perfect set. The smallest such ordinal $\alpha _0$ is called the *Cantor-Bendixson rank* of *A*.

In particular, $H^{\alpha _0}(A)=\emptyset$ for some countable ordinal $\alpha _0$ iff *A* is countable.

### Proof of Theorem 15

Let $f:M\rightarrow Y$ be a continuous function which is intrinsically Lipschitz continuous on $E:=M\setminus \Theta$. Denote by *L* the Lipschitz constant of *f*. Let *x*, *y* in *M* and let $\varepsilon >0$.

Since $\Theta$ is permeable, there exists a path $\gamma :[a,b]\rightarrow M$ from *x* to *y* with $\ell (\gamma )<\rho _M(x,y)+\varepsilon$ and such that $A_\gamma :=\overline{\{\gamma (t):t\in [a,b]\}\cap \Theta }$ is countable.

Invoking Lemma 5, we may assume that $\gamma$ is injective. Furthermore we may and will assume that $\forall t\in [a,b]:\ell (\gamma |_{[0,t]})=t$ and, in particular, $a=0,b=\ell (\gamma )$.

If we can show that the map $f\circ \gamma :[0,b]\rightarrow Y$ is *L*-Lipschitz continuous, then we are done, because then

$$\begin{aligned} d_Y\big (f(x),f(y)\big )=d_Y\big (f\circ \gamma (0),f\circ \gamma (b)\big )\le L b=L\ell (\gamma )<L(\rho _M(x,y)+\varepsilon )\,. \end{aligned}$$

Let $A:=\gamma ^{-1}(A_\gamma )\subseteq [0,b]$. Then *A* is closed since $\gamma$ is continuous. We start by showing that if $C_0$ is a connected component of $[0,b]\setminus A$ (clearly, $C_0$ is an interval with non-empty interior), then $f\circ \gamma$ is *L*-Lipschitz on $\overline{C_0}$. Indeed, let $r,s\in C_0^\circ$ with $r<s$. Since $[r,s]\cap A= \emptyset$, the restricted arc $\gamma |_{[r,s]}$ is an arc in *E* and therefore $\rho _E(\gamma (r),\gamma (s))\le \ell (\gamma |_{[r,s]})= (s-r)$ . Since *f* is intrinsically *L*-Lipschitz continuous on *E*,

1 $$\begin{aligned} d_Y\big (f\circ \gamma (r),f\circ \gamma (s)\big )\le L\rho _E(\gamma (r),\gamma (s))\le L(s-r)\,. \end{aligned}$$

By the continuity of *f*, Eq. (1) also holds for $r,s\in \overline{C_0}$.

Next let $C_1$ be a connected component of $[0,b]\setminus H(A)$. If $r,s\in C_1^\circ$ with $r<s$, then, since [*r*, *s*] does not contain an accumulation point of *A*, there exist $t_1,\ldots ,t_n\in [0,b]$ such that $t_1<\ldots <t_n$ and $[r,s]\cap A=\{t_1,\ldots ,t_n\}$. Set $t_0:=r$, $t_{n+1}:=s$. Then for every $k\in \{1,\dotsc ,n+1\}$ the interval $(t_{k-1}, t_{k})$ does not contain a point of *A*, so

$$\begin{aligned} d_Y\big (f\circ \gamma (t_{k-1}),f\circ \gamma (t_k)\big )\le L(t_k-t_{k-1})\,, \end{aligned}$$

and therefore

2 $$\begin{aligned} d_Y\Big (f\circ \gamma (r),f\circ \gamma (s)\Big )&\le \sum _{j=1}^{n+1} d_Y\Big (f\circ \gamma (t_{j-1}),f\circ \gamma (t_{j})\Big ) \nonumber \\&\le L \sum _{j=1}^{n+1} \big (t_j-t_{j-1}\big )= L (s-r)\,. \end{aligned}$$

That is,

3 $$\begin{aligned} d_Y\big (f\circ \gamma (r),f\circ \gamma (s)\big )\le L(s-r)\,. \end{aligned}$$

and by the continuity of *f*, Equation (3) also holds for $r,s\in \overline{C_1}$.

We proceed by a transfinite induction argument, where the base case has already been dealt with. The induction hypothesis is that for an ordinal $\alpha$, all ordinals $\beta <\alpha$ and every connected component $C_\beta$ of $[0,b]\setminus H^\beta (A)$, we have that $f\circ \gamma$ is *L*-Lipschitz continuous on $\overline{C_\beta }$. In order to perform the induction step, we need to show that this property extends to $\alpha$, that is, for every connected component $C_\alpha$ of $[0,b]\setminus H^\alpha (A)$, $f\circ \gamma$ is *L*-Lipschitz continuous on $\overline{C_\alpha }$.

If $\alpha$ is not a limit ordinal, we can use the same argument as in the step from the ordinal 0 to the ordinal 1.

Now assume, that $\alpha$ is a limit ordinal. Let $r,s\in C_\alpha ^\circ$, $r<s$. There exists an ordinal $\beta _0<\alpha$ such that $[r,s]\cap H^{\beta _0}(A)=\emptyset$ : Otherwise, there is an increasing sequence $(\beta _n)_{n\in {{\mathbb {N}}}}$, $\beta _n<\alpha$ for all *n*, and a sequence $(t_n)_{n\in {{\mathbb {N}}}}$, $t_n\in H^{\beta _n}(A)\cap [r,s]$ with $\lim _{n\rightarrow \infty } t_n\in H^\alpha (A)$. But this is impossible, since $[r,s]\subseteq C_\alpha ^\circ$ and $C_\alpha ^\circ \cap H^\alpha (A)=\emptyset$, so that $\inf \{|u-v|:u\in H^\alpha (A),v\in [r,s])>0$.

But $[r,s]\cap H^{\beta _0}(A)=\emptyset$ implies that $[r,s]\subseteq \overline{C_{\beta _0}}$ for some connected component $C_{\beta _0}$ of $[0,b]\setminus H^{\beta _0}(A)$. By the induction hypothesis, $f\circ \gamma$ is *L*-Lipschitz on $\overline{C_{\beta _0}}$, and we get Eq. (3).

We are ready to finish the proof. By Theorem 16 there exists a countable ordinal $\alpha _0$ such that $H^{\alpha _0}(A)=\emptyset$. But then $[0,b]\setminus H^{\alpha _0}(A)=[0,b]$ is the only connected component and therefore $f\circ \gamma$ is *L*-Lipschitz continuous on [0, *b*]. $\square$

### Remark 17

Note that the inequalities in (2) cannot be generalized in a straightforward way to a related notion of “intrinsically Hölder continuous”.

### Remark 18

1. Theorem 15 generalizes Lemma 3.6 in [18]. In the latter it is assumed that the exception set is a finitely permeable submanifold of ${{\mathbb {R}}}^d$.
2. Theorem 15 and its proof should also be compared with the results [9, Theorem 2.5 and Proposition 2.2], which together imply the following: Let *I* be a real interval, $f : I\rightarrow {{\mathbb {R}}}$ a function and $E\subseteq I$. If
  *E* has no perfect subsets,
  $$f : I\rightarrow {{\mathbb {R}}}$$ is continuous,
  the pointwise Lipschitz constant of *f* is bounded by a constant *C* at every point of $I\setminus E$.
  Then *f* is *C*-Lipschitz on *I*. This result is obviously also related to our 1-dimensional permeability criterion, Theorem 23, below.

We have two immediate corollaries of Theorem 15:

### Corollary 19

Let *M* be a *C*-quasi-convex space and let $\Theta \subseteq M$ be permeable. Then every continuous function $f:M\rightarrow Y$ into a metric space $(Y,d_Y)$, which is intrinsically *L*-Lipschitz continuous on $E=M\setminus \Theta$, is *CL*-Lipschitz continuous on the whole of *M* (i.e., with respect to *d*).

### Corollary 20

Let *M* be a length space and let $\Theta \subseteq M$ be permeable. Then every continuous function $f:M\rightarrow Y$ into a metric space $(Y,d_Y)$, which is intrinsically *L*-Lipschitz continuous on $E=M\setminus \Theta$, is *L*-Lipschitz continuous on the whole of *M* (i.e., with respect to *d*).

For example, Corollary 20 can be applied for $M={{\mathbb {R}}}^d$ with the euclidean metric, which is a length space. We will take a deeper look at this example in Sect. 3.

The next result states that, if a function is intrinsically Lipschitz except on a closed permeable set, then the Lipschitz constant does not change when one enlarges the exception set to another permeable set.

### Proposition 21

Let $\Theta _0\subseteq \Theta \subseteq M$ with $\Theta$ permeable and $\Theta _0$ closed. Let $f:M\rightarrow Y$ be intrinsically Lipschitz except on $\Theta _0$. Then *f* is intrinsically Lipschitz except on $\Theta$ and

$$\begin{aligned} \sup _{x,y\in M\setminus \Theta }\frac{d_Y(f(x),f(y))}{\rho _{M\setminus \Theta }(x,y)} =\sup _{x,y\in M\setminus \Theta _0}\frac{d_Y(f(x),f(y))}{\rho _{M\setminus \Theta _0}(x,y)} \end{aligned}$$

### Proof

Let $N=M\setminus \Theta _0$. By assumption, *f* is intrinsically Lipschitz continuous on *N*. As $\Theta \supseteq \Theta _0$, *f* is also intrinsically Lipschitz continuous on $M\setminus \Theta =N\setminus (\Theta \setminus \Theta _0)$, and $\Theta \setminus \Theta _0$ is permeable in *N*, by Proposition 14. Therefore the assertion follows from Theorem 15. $\square$

### Example 22

Consider $(M,d):=({{\mathbb {R}}}^2,|.|)$ and $\Theta :={{\mathbb {Q}}}^2$. Then $\rho ^{{{\mathbb {Q}}}^2}_{{{\mathbb {R}}}^2}(x,y)=|x-y|$ for all $x,y\in {{\mathbb {R}}}^2$. By Corollary 20, every intrinsically Lipschitz function on *M* with exception set ${{\mathbb {Q}}}^2$, which is continuous on ${{\mathbb {R}}}^2$, is Lipschitz on the whole of ${{\mathbb {R}}}^2$.

We now show that in the one-dimensional euclidean case the permeable sets $\Theta$ are precisely those for which every function is Lipschitz iff it is continuous and intrinsically Lipschitz with exception set $\Theta$. Note that for subsets of ${{\mathbb {R}}}$ permeability is equivalent to having countable closure.

### Theorem 23

Let $\Theta \subseteq {{\mathbb {R}}}$. Then $\Theta$ has countable closure if and only if for all intervals $I\subseteq {{\mathbb {R}}}$ and all functions $f:I\rightarrow {{\mathbb {R}}}$ the properties

- *f* is intrinsically Lipschitz continuous with exception set $\Theta$,
- *f* is continuous,

imply that *f* is Lipschitz continuous on *I*.

### Proof

The only if part follows from Corollary 20.

For the “if part” assume to the contrary that $\Theta$ has uncountable closure. Then ${\overline{\Theta }}=A\cup P$, where *A* is countable and *P* is perfect by the Cantor-Bendixson theorem, [16, Theorem 6.4]. Hence, *P* contains a homeomorphic copy of the Cantor set by [16, Theorem 6.5]. Let *f* be the corresponding Cantor staircase function. Then *f* is continuous and *f* is constant on every connected component of ${{\mathbb {R}}}\setminus \Theta$. But the Cantor staircase function is not Lipschitz. $\square$

The following proposition by Tapio Rajala^2^ (personal communication) connects consequences of permeability and the statement of Corollary 20, that intrinsically Lipschitz continuity for a given exception set implies the overall Lipschitz continuity. It should also be compared to Proposition 13, which states that subsets of permeable sets are permeable.

### Proposition 24

Let $\Theta \subseteq M$ have the property that every function $f:M \rightarrow Y$ which is continuous and intrinsically (*L*-)Lipschitz continuous with exception set $\Theta$ is (*L*-)Lipschitz continuous.

Then every subset $\Theta _0$ of $\Theta$ enjoys the same property.

### Proof

If a function $f:M\rightarrow Y$ is (*L*-)Lipschitz continuous with respect to $\rho _{M\setminus \Theta _0}$, then, as $\rho _{M\setminus \Theta _0}\le \rho _{M\setminus \Theta }$, *f* is (*L*-)Lipschitz continuous with respect to $\rho _{M\setminus \Theta }$ implying (*L*-)Lipschitz continuity of *f* on the whole of *M*. $\square$

### Remark 25

With regard to Corollary 20, Theorem 23 and Proposition 13, one may ask the following interesting question:

*In* ${{\mathbb {R}}}^d$, *are the permeable subsets* $\Theta$ *precisely those for which every function is* *L*-*Lipschitz iff it is continuous and intrinsically* *L*-*Lipschitz with exception set* $\Theta$?

The following example, proposed by Tapio Rajala (personal communication), shows that one may not reduce ‘*L*-Lipschitz’ to merely ‘Lipschitz’ in the above question: Consider the set $\Theta =([0,1]\setminus {\mathbb {Q}})^2\subseteq M=[0,1]^2$. Then the intrinsic metric $\rho _{M\setminus \Theta }$ is given by the 1-distance, see [15, Proposition 3.6]. Hence, if a function is continuous and *L*-Lipschitz with respect to $\rho _{M\setminus \Theta }$, then it will be continuous and $\sqrt{2}L$-Lipschitz with respect to the euclidean metric on $[0,1]^2$, and therefore Lipschitz. On the other hand, $\Theta$ is not permeable, which we show in the subsequent proposition.

### Proposition 26

Let $M=[0,1]^2$ be endowed with the euclidean distance and let $\Theta =([0,1]\setminus {\mathbb {Q}})^2$. Then $\Theta$ is not permeable.

### Proof

Assume that $\gamma$ is a path from (0, 0) to (1, 1) of length $\ell (\gamma )<\sqrt{2}+\varepsilon$. As we can always shorten parts of $\gamma$, where its first component is not monotonically increasing, by a vertical line with rational first component, it suffices to assume $\gamma$ to be given by a function $\tilde{\gamma }:[0,1]\rightarrow [0,1]$ of arc length smaller than $\sqrt{2}+\varepsilon$. Similarly, one may replace intervals, where ${\tilde{\gamma }}$ is strictly decreasing, by a horizontal segment with rational function value. So we may assume ${\tilde{\gamma }}$ to be monotonically increasing. As monotonically increasing function, its derivative exists almost everywhere in [0, 1]. The derivative can not be zero almost everywhere, for then the function’s arc length were 2 which is not smaller than $\sqrt{2}+\varepsilon$ for arbitrary $\varepsilon$, see e.g., [10, Theorem 4]. Thus there must be a set *A* with $\lambda (A)>0$, where $\tilde{\gamma }'(x)>0$ for all $x\in A$. Since the derivatives are positive on *A*, it follows that $\tilde{\gamma }(x)<\tilde{\gamma }(x')$ for $x<x',\, x,x'\in A$. Since $M\setminus \Theta = \left( ([0,1]\cap {\mathbb {Q}})\times ([0,1]\setminus {\mathbb {Q}})\right) \cup \left( ([0,1]\setminus {\mathbb {Q}})\times ([0,1]\cap {\mathbb {Q}})\right)$,

4 $$\begin{aligned} \mathrm {Graph}(\tilde{\gamma }\!\!\mid _A)\cap (M\setminus \Theta )=\bigcup _{x\in A\cap {\mathbb {Q}}}\left\{ (x,\tilde{\gamma }(x))\right\} \cup \bigcup _{y\in {\mathbb {Q}}\cap {\tilde{\gamma }}(A)}\left\{ \big ((\tilde{\gamma }\!\!\mid _A)^{-1}(y),y\big )\right\} . \end{aligned}$$

As *A* is uncountable, $\mathrm {Graph}(\tilde{\gamma }\!\!\mid _A)$ has uncountably many values in the first component, but $\mathrm {Graph}(\tilde{\gamma }\!\!\mid _A)$ intersects the first union of (4) in countably many points. In the same way, the second union intersects $\mathrm {Graph}(\tilde{\gamma }\!\!\mid _A)$ only in countably many points. Therefore $\mathrm {Graph}(\tilde{\gamma }\!\!\mid _A)\subseteq \mathrm {Graph}(\tilde{\gamma })$ intersects $\Theta$ in uncountably many points. We conclude that $\Theta$ is not permeable. $\square$

We have shown in Proposition 12 that permeable sets cannot contain interior points. It therefore makes sense to study subsets $\Theta \subseteq {{\mathbb {R}}}^d$ that have no interior points relative to ${{\mathbb {R}}}^d$, and in the next section we further specialize to submanifolds of ${{\mathbb {R}}}^d$ with dimension strictly smaller than *d*.

## Sub-manifolds of ${{\mathbb {R}}}^d$ as Permeable Sets

### Definition 27

Let $d,m,k\in {{\mathbb {N}}}\cup \{0\}$, $m < d$, and let $\Theta \subseteq {{\mathbb {R}}}^d$. We say $\Theta$ is an *m*-dimensional $C^k$-*submanifold* of ${{\mathbb {R}}}^d$ iff for every $\xi \in \Theta$ there exist open sets $U,V\subseteq {{\mathbb {R}}}^d$ and a $C^k$-diffeomorphism $\Psi :V\rightarrow U$ such that $\xi \in U$ and for all $y=(y_1,\dots ,y_d)\in V$ it holds $\Psi (y)\in \Theta \Longleftrightarrow y_{m+1}=\dots =y_d=0$. In the case where $k=0$, by a $C^0$-diffeomorphism we mean a homeomorphism, and we also call $\Theta$ a *topological submanifold* (top-*submanifold) *.

### Definition 28

A top-submanifold of ${{\mathbb {R}}}^d$ of dimension $m<d$ is called *Lipschitz* or of *class* lip if the mappings $\Psi , \Psi ^{-1}$ from Definition 27 are Lipschitz continuous on every compact subset of their respective domain.

### Corollary 29

The class of Lipschitz submanifolds contains those that possess continuously differentiable mappings $\Psi ,\Psi ^{-1}$ (class $C^1$) as well as those having mappings $\Psi ,\Psi ^{-1}$ that are continuous and piecewise linear, resp. piecewise differentiable, on subdivisions of *U*, *V* into polyhedra (class pl, resp. class pdiff).

Before we state the main result of this section, we prove a preparatory lemma.

### Lemma 30

Let $\Theta \subseteq {{\mathbb {R}}}^d$ be a Lebesgue-nullset. Then for all $x,y\in {{\mathbb {R}}}^d$ and all $\varepsilon >0$ there exists a polygonal chain $\gamma :[a,b]\rightarrow {{\mathbb {R}}}^d$ such that $\ell (\gamma )<\Vert y-x\Vert +\varepsilon$ and $\{t\in [a,b]:\gamma (t)\in \Theta \}$ is a Lebesgue-nullset.

### Proof

For the case $d=1$ there is nothing to prove. If $d>1$, consider the $(d-1)$-dimensional ball *B* with center $\frac{1}{2}(x+y)$ and radius $\frac{1}{2}\sqrt{(\Vert y-x\Vert +\frac{\varepsilon }{2})^2-\Vert y-x\Vert ^2}$ that lies in the hyperplane orthogonal to $x-y$ and passes through $\frac{1}{2}(x+y)$. Then the convex hull of $B\cup \{x,y\}$, which we denote by *C*, is a *d*-dimensional double cone and, since $\Theta$ is a Lebesgue-nullset, we have $\lambda ^d(C\cap \Theta )=0$, where $\lambda ^d$ is the Lebesgue-measure on ${{\mathbb {R}}}^d$.

By Fubini’s theorem,

$$\begin{aligned} 0&=\lambda ^d(C\cap \Theta )\\&=\frac{1}{2}\Vert x-y\Vert \int _B \int _{[0,1]} \Big (1_\Theta ((1-t)x+t z)+1_\Theta ((1-t)y+t z)\Big ) (1-t)^{d-1} dt\, dz\,. \end{aligned}$$

From this we conclude that

$$\begin{aligned} \int _0^1 \Big (1_\Theta ((1-t)x+t z)+1_\Theta ((1-t)y+t z)\Big ) (1-t)^{d-1} dt =0\,, \end{aligned}$$

for almost all $z\in B$. We may choose one such $z\in B$, and for this we have $1_\Theta ((1-t)x+t z)+1_\Theta ((1-t)y+t z)=0$ for almost all $t\in (0,1)$. Thus the proof is finished. $\square$

### Theorem 31

Let $\Theta \subseteq {{\mathbb {R}}}^d$ be a lip-submanifold which in addition is a closed subset of ${{\mathbb {R}}}^d$. Then for all $x,y\in {{\mathbb {R}}}^d$ and all $\varepsilon >0$ there exists a path $\gamma :[a,b]\rightarrow {{\mathbb {R}}}^d$ from *x* to *y* with $\ell (\gamma )<\Vert x-y\Vert +\varepsilon$ and such that $\gamma \big ([a,b]\big )\cap \Theta$ is finite. Therefore, $\Theta$ is finitely permeable and hence permeable.

### Proof

Let $$x,y\in {{\mathbb {R}}}^d$$ and $$\varepsilon >0$$.

*Step 1* Note that, since $\Theta$ is a Lipschitz topological submanifold and is thus locally the Lipschitz image of a Lebesgue-nullset, it is itself a Lebesgue-nullset (with respect to ${{\mathbb {R}}}^d$) as bi-Lipschitz homeomorphisms pertain measurability of sets and therefore preserve Lebesgue-nullsets, see, e.g., [27, Lemma 7.25]. By virtue of Lemma 30 we may thus restrict our considerations to the case where $F:=\big \{t\in [0,1]:(1-t)x+ty\in \Theta \big \}$ has Lebesgue measure 0 (in [0, 1]).

If *F* is finite, then we are done. Otherwise assume first that $x\notin \Theta$. Since $\Theta$ is a closed set, we can find $z\in \Theta$ such that the line segment $\gamma _1$ connecting *x* and *z* intersects $\Theta$ precisely in *z*. If we can find a path $\gamma _2:[a,b]\rightarrow {{\mathbb {R}}}^d$ from *z* to *y* with $\ell (\gamma _2)<\Vert y-z\Vert +\varepsilon$ and such that $\gamma _2\big ([a,b]\big )\cap \Theta$ is finite, then the concatenation $\gamma$ of the paths $\gamma _1$ and $\gamma _2$ is a path with the required properties and we are done. Thus we may assume that $x\in \Theta$ and, by the same argument, that $y\in \Theta$.

*Step 2* Write $g:[0,1]\rightarrow {{\mathbb {R}}}^d$, $g(t):=(1-t)x+ty$. Since $\big \{ g(t) :t\in F\big \}\subseteq \Theta$ is compact, there exist finitely many $t_1,\ldots ,t_n\in F$ and bounded open environments $U_j$ of $g(t_j)$, $V_j$ of 0 and $\Psi _j:V_j\rightarrow U_j$ bi-Lipschitz such that for all $z=(z_1,\dots ,z_d)\in V_j$ it holds $\Psi _j(z)\in \Theta \Longleftrightarrow z_{m+1}=\dots =z_d=0$.

*F* is a non-empty closed subset of [0, 1], so we can write

$$\begin{aligned}{}[0,1]\setminus F=\bigcup _{k=1}^\infty (a_k,b_k)\,, \end{aligned}$$

where the right hand side is a disjoint union and, since $\lambda (F)=0$, $\sum _{k=1}^\infty (b_k-a_k)=1$. Now for every $K\in {{\mathbb {N}}}$ there exist $c_0,\ldots ,c_K,d_0,\ldots ,d_K$ with

5 $$\begin{aligned} \Big (\bigcup _{k=1}^K (a_k,b_k)\Big )^c =\bigcup _{k=0}^{K} [c_k,d_k] \supseteq F\,, \end{aligned}$$

where $[c_0,d_0],\ldots ,[c_K,d_K]$ are again disjoint. We may assume that *K* is large enough to guarantee that for every interval $[c_k,d_k]$ there exists $j\in \{1,\ldots ,n\}$ such that $g( [c_k,d_k])\subseteq U_j$. In addition, we may assume that for every *j* the functions $\Psi _j$ and $\Psi ^{-1}_j$ are Lipschitz with common constant $L_j$. If we can find, for every $k=0,\ldots ,K$, a path $\gamma _k:[0,1]\rightarrow {{\mathbb {R}}}^d$ from $g(c_k)$ to $g(d_k)$ with $\ell (\gamma _k)<\Vert g(d_k)-g(c_k)\Vert +\frac{\varepsilon }{K+1}$ and such that $\gamma _k([c_k,d_k])\cap \Theta$ is finite, then we can construct a path with the required properties. We may therefore concentrate on the case where the whole of *g*([0, 1]) is contained in a single $U_j$, which we will do in Step 3.

*Step 3* Write $U:=U_j$, $V:=V_j$, $\Psi :=\Psi _j$, $L:=L_j$. Since $\Psi$ and $\Psi ^{-1}$ are Lipschitz continuous with constant *L*, we have for every finite collection of paths $\eta _1,\ldots ,\eta _N$ in *U*

$$\begin{aligned} \sum _{n=1}^N \ell (\Psi ^{-1}\circ \eta _n)\le L\sum _{n=1}^N \ell (\eta _n) \,. \end{aligned}$$

for any finite collection of paths $\kappa _1,\ldots ,\kappa _N$ in *V*

$$\begin{aligned} \sum _{n=1}^N \ell (\Psi \circ \kappa _n)\le L\sum _{n=1}^N \ell (\kappa _n)\,. \end{aligned}$$

We repeat the earlier argument to get, for every $K\in {{\mathbb {N}}}$, a disjoint union of the type (5). Now we choose *K* big enough to ensure $\sum _{k=0}^K (d_k-c_k)<\frac{\varepsilon }{2 L^2 \Vert y-x\Vert }$. We write $x_k:=(1-c_k)x+c_k y$ and $y_k:=(1-d_k)x+d_k y$. For every $k\in \{0,\ldots ,K\}$, let $\kappa _k:[c_k,d_k]\rightarrow U$, $\kappa _k(t):=(1-t)x+t y$ that is, $\kappa _k$ is a parametrization of the line-segment from $x_k$ to $y_k$. Now $\kappa _0,\ldots ,\kappa _K$ is a finite collection of paths with

$$\begin{aligned}&\sum _{k=0}^K \Vert \Psi ^{-1}\circ \kappa _k(d_k)-\Psi ^{-1}\circ \kappa _k(c_k)\Vert \\&\quad \le \sum _{k=0}^K \ell (\Psi ^{-1}\circ \kappa _k)\le L\sum _{k=0}^K \ell (\kappa _k)\\&\quad \le L\sum _{k=0}^K (d_k-c_k)\Vert y-x\Vert <\frac{\varepsilon }{2 L}\,. \end{aligned}$$

Set $\ell _k:=\Vert \Psi ^{-1}\circ \kappa _k(d_k)-\Psi ^{-1}\circ \kappa _k(c_k)\Vert$. For every *k* with $\ell _k=0$ let $\eta _k$ be constant equal to $\Psi ^{-1}\circ \kappa _k(c_k)$.

Denote by $e_d$ the vector $(0,\ldots ,0,1)$. For every *k* with $\ell _k>0$ we construct a path $\eta _k:[0,2 \ell _k]\rightarrow V$ by

$$\begin{aligned} \eta _k(t)={\left\{ \begin{array}{ll} \Psi ^{-1}\circ \kappa _k(c_k)+t a e_d&{} \text {if } 0\le t \le \frac{\ell _k}{2}\,,\\ \frac{3\ell _k-2t}{2\ell _k}\Psi ^{-1}\circ \kappa _k(c_k)+\frac{2t- \ell _k}{2\ell _k}\Psi ^{-1}\circ \kappa _k(d_k)+\frac{\ell _k}{2} a e_d&{} \text {if } \frac{\ell _k}{2}\le t \le \frac{3\ell _k}{2}\,,\\ \Psi ^{-1}\circ \kappa _k(d_k)+(2\ell _k-t)a e_d&{} \text {if } \frac{3\ell _k}{2}\le t \le 2 \ell _k\,, \end{array}\right. } \end{aligned}$$

where $a\in (0,1)$ is small enough so that $\eta _k(t)\in V$ for all $t\in [0,2 \ell _k]$. By construction, $\ell (\eta _k)\le 2\ell _k$, such that

$$\begin{aligned} \sum _{k=0}^K \ell (\Psi \circ \eta _k)\le L\sum _{k=0}^K \ell (\eta _k)\le 2L\sum _{k=0}^K \ell _k< \varepsilon . \end{aligned}$$

Now define $\gamma$ as the concatenation of the following paths:

- the paths $\Psi \circ \eta _k$ from $x_k$ to $y_k$ for $k=0,\ldots ,K$;
- the line segments from $y_{k-1}$ to $x_k$ with lengths ${\hat{\ell }}_k:=\Vert x_k-y_{k-1}\Vert$ for $k=1,\ldots ,K$.

Summing up, we get for the length of $\gamma$

$$\begin{aligned} \ell (\gamma )&=\sum _{k=1}^K {\hat{\ell }}_k+\sum _{k=0}^K \ell (\Psi \circ \eta _k)\\&=\sum _{k=1}^K \Vert x_k-y_{k-1}\Vert +\sum _{k=0}^K \ell (\Psi \circ \eta _k)\\&<\Vert y-x\Vert +\varepsilon \,, \end{aligned}$$

and $\gamma$ has only finitely many intersections with $\Theta$. $\square$

### Corollary 32

Let $\Theta$ be a Lipschitz submanifold of the ${{\mathbb {R}}}^d$ which is closed as a subset.

Then every continuous function $f:{{\mathbb {R}}}^d\rightarrow {{\mathbb {R}}}$ which is intrinsically Lipschitz continuous with exception set $\Theta$ is Lipschitz continuous.

### Proof

This follows immediately from Corollary 20 and Theorem 31. $\square$

### Example 33

Consider the topologist’s sine:

$$\begin{aligned} \Theta&:=\left\{ \left( t,\sin \left( \tfrac{1}{t}\right) \right) :t\in (0,\infty )\right\} \,,\\ {\overline{\Theta }}&\;=\left\{ \left( t,\sin \left( \tfrac{1}{t}\right) \right) :t\in (0,\infty )\right\} \cup \big \{(0,s):s\in [-1,1]\big \} \,. \end{aligned}$$

It is readily checked that $\rho ^{{\overline{\Theta }}}_{{{\mathbb {R}}}^2}=\rho ^\Theta _{{{\mathbb {R}}}^2}=\rho _{{{\mathbb {R}}}^2}$, i.e., both $\Theta$ and ${\overline{\Theta }}$ are permeable. $\Theta$ is a sub-manifold of ${{\mathbb {R}}}^2$, which is not a subset of a topologically closed submanifold of the ${{\mathbb {R}}}^d$, while ${\overline{\Theta }}$ is closed, but not a submanifold. Note also, that for $x=(0,0)$, $y=(1,0)$ there is no path connecting *x* and *y* of length smaller than 2 which has a finite intersection with $\Theta$, in contrast to the case of closed Lipschitz submanifolds. Therefore $\Theta$ is permeable but not finitely permeable.

The following example shows that one cannot simply dispense with the assumption that the exception set is topologically closed.

### Example 34

Recall that the classical Cantor set *C* is the topological closure of the set

$$\begin{aligned} C_0=\left\{ \sum _{k=1}^n d_k3^{-k}: n\in {{\mathbb {N}}}, d_k\in \{0,2\}\right\} \,. \end{aligned}$$

Every element from $C_0$ is the limit of an increasing sequence in ${{\mathbb {R}}}\setminus \overline{C_0}$:

$$\begin{aligned} 0&=\lim _{j} -3^{-j}, \\ \sum _{k=1}^n d_k3^{-k}&=\lim _{j\rightarrow \infty } \left( \sum _{k=1}^n d_k3^{-k}-3^{-(j+n)}\right) ,&(n\in {{\mathbb {N}}}, d_n=2). \end{aligned}$$

Denote by $D_0$ the union of all elements of all these sequences. Then $D_0$ consists only of isolated points and $\overline{D_0}\supseteq \overline{C_0}=C$. Therefore $D_0$ is a 0-dimensional submanifold of ${{\mathbb {R}}}$ which is not permeable.

By extruding $D_0$ to $D_{d-1}:=D_0\times {{\mathbb {R}}}^{d-1}$ we get an example of a $(d-1)$-dimensional $C^\infty$-submanifold which is not permeable (and not topologically closed).

We conclude this section with more examples of permeable sets. First note that we can somewhat relax the requirement that a Lipschitz manifold be a closed subset of ${{\mathbb {R}}}^d$, since by Proposition 13 the property $\rho ^\Theta _{{{\mathbb {R}}}^d}=\rho _{{{\mathbb {R}}}^d}$ extends to subsets.

The conclusion of Theorem 31 also holds for unions of closed Lipschitz submanifolds with transversal intersections, with the following notion of *transversal intersection*:

### Definition 35

Let $\Theta _1$ and $\Theta _2$ be two $C^k$-submanifolds of the ${{\mathbb {R}}}^d$, and let $\xi \in \Theta _1\cap \Theta _2$. We say that $\Theta _1$ and $\Theta _2$ *intersect transversally* in $\xi$, iff there exist

- open sets $U,V\subseteq {{\mathbb {R}}}^d$ such that $\xi \in U$
- a $$C^k$$-diffeomorphism $$\Psi :V\rightarrow U$$
- linear subspaces $E_1$, $E_2$ with $\dim (E_j)=\dim (\Theta _j)$, $j=1,2$,

such that $$\Psi (V\cap E_j)=U\cap \Theta _j$$, $$j=1,2$$.

We say $\Theta _1$ and $\Theta _2$ *intersect transversally* iff they intersect transversally in every $\xi \in \Theta _1\cap \Theta _2$.

In the cases of a top- or lip-submanifold, one has to replace $C^k$-submanifolds and $C^k$-diffeomorphism above by the notions for the respective classes.

The proof of Theorem 31 for transversally intersecting unions of closed lip-submanifolds differs only in the construction of the paths $\eta _k$. There one has to distinguish different cases, whether both endpoints lie in different $E_1$, $E_2$ or in the same or even in both. The procedure can be extended to finitely many intersecting closed lip-submanifolds. Similar arguments yield that a topologically closed lip-submanifold with boundary is permeable.

### Remark 36

As unions of affine hyperplanes exhibit transversal intersections, they are permeable. Hence piecewise Lipschitz functions in the sense of Definition 3 have permeable exception sets.

### Remark 37

Let $f:{{\mathbb {R}}}\rightarrow {{\mathbb {R}}}$ be Hölder continuous with Hölder exponent $\alpha \in (0,1)$ and let

$$\begin{aligned} \Theta :=\left\{ (t,f(t)):t\in {{\mathbb {R}}}\right\} \,. \end{aligned}$$

An interesting question is: For which $\alpha$, if for any, is $\Theta$ permeable?

The motivation for this example comes from the standard result from probability theory that almost every path of an $({\mathscr {F}}_t)_{[0,1]}$-Brownian motion *B* on some probability space $(\Omega ,{\mathscr {F}},({\mathscr {F}}_t)_{t\in [0,1]},P)$ is Hölder continuous with exponent $<\frac{1}{2}$. The graph of such a path constitutes a top-submanifold (with boundary), but not a lip-submanifold^3^, so Theorem 31 does not apply. A strong hint that Brownian paths might not be permeable is the following: Consider a bounded and progressively measurable process $H:\Omega \times [0,1]\rightarrow {{\mathbb {R}}}$ and an equivalent change of measure from *P* to ${\hat{P}}$ such that ${\hat{B}}_t:=B_t-\int _0^t H_sds$ defines a Brownian motion under ${\hat{P}}$ (this change of measure exists by Girsanov’s theorem). Now the set $\{t\in [0,1]:{\hat{B}}_t=0\}$ is uncountable with probability 1 under ${\hat{P}}$. Therefore

$$\begin{aligned} P\left( \left\{ t\in [0,1]:B_t=\int _0^t H_s ds\right\} \text { is countable } \right) =0\,. \end{aligned}$$

From that we conclude that, for a given $\omega \in \Omega$ the graph of the function $g:[0,1]\rightarrow {{\mathbb {R}}}$ with $g(t):=\int _0^t H_s(\omega )ds$ has uncountable intersection with the graph of $B(\omega )$ almost surely. A further hint in this direction is Theorem 1.5 in [3]. It states that for every continuous function $g:[0,1]\rightarrow {{\mathbb {R}}}$ the zeros of $B-g$ have Hausdorff dimension at least $\frac{1}{2}$ with positive probability. On the other hand, Theorem 1.2 in [3] states that there exists a function *g*, which is Hölder continuous with exponent smaller $\frac{1}{2}$ such that $B-g$ has isolated zeros with positive probability (nothing is said there about the length of the graph of *g*). See also the related questions in [24, Open Problem (1)].

## Footnotes

1 By a polyhedron we mean the intersection of finitely many affine half spaces in ${{\mathbb {R}}}^d$.

2 University of Jyvaskyla, Finland

3 To see this, assume that there is a bi-lip mapping which locally maps the path to the *x*-axis. Then the path from *x* to $x+\delta$, $\delta >0$ sufficiently small, would be mapped back to a finite length arc in the Brownian path, which is impossible.
